# An Overview of the Potential Antineoplastic Effects of Casticin

**DOI:** 10.3390/molecules25061287

**Published:** 2020-03-12

**Authors:** Shanaya Ramchandani, Irum Naz, Jong Hyun Lee, Muhammad Rashid Khan, Kwang Seok Ahn

**Affiliations:** 1 Department of Pharmacology—Biomedicine, The University of Melbourne, Parkville, VIC 3010, Australia; ramchandanishanaya@gmail.com; 2 Department of Biochemistry, Quaid-i-Azam University, Higher Education Commission of Pakistan, Islamabad 44000, Pakistan; irumnaz@bs.qau.edu.pk; 3 Department of Science in Korean Medicine, College of Korean Medicine, Kyung Hee University, #47, Kyungheedae-gil, Dongdaemoon-gu, Seoul 130–701, Korea; mirue88@nate.com

**Keywords:** cancer, casticin, flavonoid, apoptosis, PI3K/Akt, NF-κB, STAT3, FOXO3a/FoxM1

## Abstract

Cancer persists as one of the leading causes of deaths worldwide, contributing to approximately 9.6 million deaths per annum in recent years. Despite the numerous advancements in cancer treatment, there is still abundant scope to mitigate recurrence, adverse side effects and toxicities caused by existing pharmaceutical drugs. To achieve this, many phytochemicals from plants and natural products have been tested against cancer cell lines in vivo and in vitro. Likewise, casticin, a flavonoid extracted from the *Vitex* species, has been isolated from the leaves and seeds of *V. trifolia* and *V. agnus-castus*. Casticin possesses a wide range of therapeutic properties, including analgesic, anti-inflammatory, antiangiogenic, antiasthmatic and antineoplastic activities. Several studies have been conducted on the anticancer effects of casticin against cancers, including breast, bladder, oral, lung, leukemia and hepatocellular carcinomas. The compound inhibits invasion, migration and proliferation and induces apoptosis (casticin-induced, ROS-mediated and mitochondrial-dependent) and cell cycle arrest (G0/G1, G2/M, etc.) through different signaling pathways, namely the PI3K/Akt, NF-κB, STAT3 and FOXO3a/FoxM1 pathways. This review summarizes the chemo-preventive ability of casticin as an antineoplastic agent against several malignancies.

## 1. Introduction

Cancer is the second-most common cause of death globally, responsible for 9.6 million fatalities in 2018 alone, i.e., one in six deaths, globally [1,2,3,4,5,6]. Approximately 17 million people are diagnosed with cancer every year, of which, 70% of deaths occur in lower-middle- and lower-class income families [1]. As a result, it is a primary objective of research scientists and oncologists to make frequent advancements in cancer research [7,8,9,10,11]. Although there are already effective treatments in place, certain cancers, such as ovarian and bladder cancers, have high recurrence rates of 50–85%, whereas breast and kidney cancers have comparatively low recurrence rates, between 5–15% [12]. Additionally, a substantial minority of patients live with the disabling fear of recurrence, negatively impacting both their mental health and quality of life [1].

Cancer is characterized by the uncontrolled growth of abnormal cells generating a malignant neoplasm (tumor), which have the potential to invade, migrate or metastasize to various other parts of the body. The properties of cancerous cells proposed by Hanahan and Weinberg can be described by its hallmarks, namely: selective growth, proliferative advantage, altered stress response that favors overall survival, promoting angiogenesis, invasion, cell movement from the primary site, metastasis, metabolic rewiring, an abetting environment and immune modulation [13]. Several risk factors play a critical role in stimulating these hallmarks. These factors can be environmental, such as tobacco intake, exposure to UV radiation, excess consumption of alcohol and red meat, and occupational hazards, or genetic, including mutations and immune conditions. Some risk factors, such as excess body weight, can be a combination of both genetic predispositions and environmental influences [14,15]. Often times, two or more risk factors act in conjunction to initiate and promote the proliferation of cancerous cells. Cancers caused by modifiable risk factors can be prevented by eliminating or mitigating the consumption and/or exposure to the sources. Vaccinations such as human papillomavirus (HPV) and hepatitis B virus (HBV) can prevent cancers caused by infectious agents. Finally, regular screenings for lesions causing colorectal and cervical cancers are crucial in cancer prevention [14].

The severity and metastatic ability of the cancer determines the treatment the patient receives. Surgery, radiation and chemotherapy are the most common means of treating cancer. Chemotherapy and radiation, however, do not only kill rapidly proliferating cancer cells but can often target healthy, regularly dividing cells, causing hair loss and depletion of the digestive lining. The side effects of these treatments can emerge decades after the treatment. Plants, and compounds extracted from plants, have been used therapeutically since ancient times against ailments [16,17,18]. In recent years, numerous natural products have been tested against cancer—in vitro, in vivo and clinically—creating a scope for treatments without severe side effects. Natural drugs such as Zerumbone—also known as bitter ginger or shampoo ginger—derived from the *Zingiber zerumbet* Smith plant species has elicited antitumor properties while pre-existing as an herbal folk medicine and food-flavoring agent [19]. Celastrol is another plant triterpene that is known to inhibit the proliferation of tumor cells and induce apoptosis (cell death) through the suppression of multiple oncogenic pathways [18,20].

Likewise, casticin, a compound extracted from the *Fructus Viticis* species (family: *Verbenaceae),* has been used for millennia as a form of traditional Chinese medicine as an analgesic to alleviate premenstrual symptoms in women, migraines and rheumatic pains. It has anti-inflammatory, antiasthmatic and antiangiogenic properties [21]. There are over 250 different species of Vitex shrubs [22,23], of which, many have medicinal uses, though the antineoplastic effects have only been evaluated in the *Vitex trifolia*, *V. negundo* and *V. agnus-castus* species thus far [24]. Promising studies of the effects of casticin have been conducted on lung, breast, colon and prostate cancer cell lines—the four most common types of cancer worldwide [14]. The effect of this flavonoid has also been tested on other cancers, including leukemia, bladder, esophageal and ovarian cancer. Additional upstream and downstream regulatory proteins are targeted by the casticin molecule in several oncogenic signaling pathways, such as the PI3K/Akt, nuclear factor kappa-B cell (NF-κB), STAT3, c-Met, etc. This review provides an updated compilation of the antineoplastic effects of casticin on various malignancies.

## 2. Chemistry

**Figure 1:**
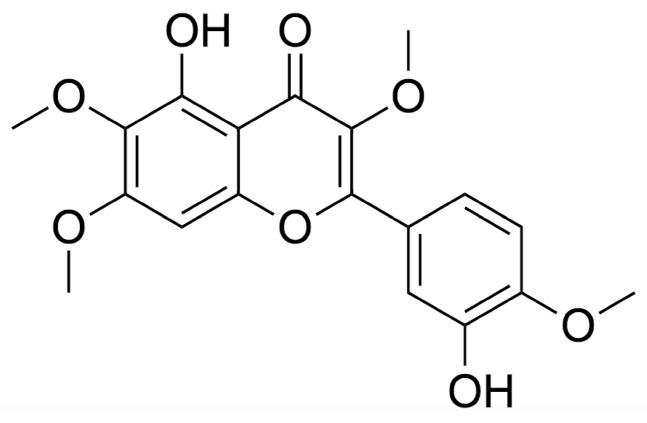
Molecular structure of casticin.

Casticin, also referred to as vitexicarpin or casticine [25], is a nonisotopic tetramethoxyflavone with three phenyl rings, an ortho catechol moiety, an alkene group, two hydroxyl groups and four methoxy groups. It has the chemical formula C19H18O8, a molecular mass of 374.3 g/mol and a complexity of 576 [26,27]. Commercially, the compound appears as yellow crystals and has a 98% purity for laboratory use [25]. It has been documented that many plants—like those from the Vitex species—contain flavonoid properties; these properties induce cell growth inhibition and apoptosis (cell death) induction [28]. Moreover, polymethoxyflavones have a strong cytotoxicity and are constituents of chemo-preventive and chemotherapy drugs. C-3′ and C-5 hydroxy, as well as C-3 and C-4′ methoxy groups in the casticin molecule, consist of significant antiproliferative activity, resulting in a proliferative disadvantage—a desirable characteristic of anticancer drugs [26]. Casticin is also insoluble in water and a weak acidic compound, making it suitable for the slightly alkaline (pH 7.35–7.45) bodily conditions in humans [29]. The molecular structure of the casticin molecule is shown in Figure 1. Specifically, casticin is isolated from the leaves [30,31,32] and fruits [30,33,34] of *V. trifolia* and *V. negundo*. The compound can also be isolated from various other plant species, including *Psiada trinervia* [35], *Daphne genkwa* [36] and *Pluchea quitoc* [37], to name a few.

## 3. Antineoplastic Effects of Casticin

In recent decades, it has been established that cancer is a product of the upregulation or downregulation of components (effectors, receptors, proteins, etc.) of multiple cellular pathways acting together. This provokes the hallmarks of cancer, enabling the cells to proliferate, invade, migrate or metastasize [13]. As a result, compounds such as casticin, which has a strong cytotoxicity, possess great potential to block these components. It also minimizes the need for treatments such as chemotherapy and radiation, which have been the primary methods to shrink a neoplasm thus far.

### 3.1. Biological Techniques

Various biological assays can be used to test the effect of a drug against the hallmarks of cancer. The MTT assay relies on the cellular reduction of tetrazolium salts to form formazan crystals to assess cell viability. As used to test cell viability in oral squamous cell carcinomas (OCSS) mentioned below, the assay is entirely dependent on mitochondrial respiration and, incidentally, analyses the capacity or cellular energy of a cell [38]. The annexin V FITC apoptosis assay assesses the change in a membrane structure upon the addition of a drug. Phospholipid asymmetry exposes the protein phosphatidylserine (PS) on the membrane surface. The annexin V dye binds to PS, exposed on dead cells, highlighting the cells that are necrotic, apoptotic or in the early apoptosis stage. Likewise, the cell cycle arrest apoptosis assay utilizes propidium iodide (PI) dye to stain the fixed cells whose kinases have phosphorylated [39,40,41]. Cell cycle arrest is a point in the cell cycle where cells no longer have the ability to duplicate or divide [40]. Migration and invasion assays use chambers to investigate the migratory and invasion abilities of cells across an extracellular matrix to test for metastatic and angiogenic properties of cancer cells [42].

Besides these relatively simple biological assays, techniques such as ELISA, RT-PCR, RNA interference, Western blot analysis and statistical analysis are used to derive detailed findings and results that can be used in pharmacological applications. ELISA, or histone/DNA enzyme-linked immunosorbent assay, is utilized to detect the presence of a ligand using antibodies to test the protein content in a cancerous cell line or tumor xenograft. A color change is used to detect a matching antibody-antigen pair, which is linked to a substrate-containing compound and an enzyme, staining the products formed [43]. ELISA is used to analyze the downregulation or upregulation of numerous proteins, which will be discussed to explain molecular mechanisms in this review. Reverse transcriptase polymerase chain reaction (RT-PCR) is used to encode gene mechanisms, such as FoxM1, amplifying the gene and searching for any mutations within the DNA [44]. The integrity is assessed using 2% agar gel electrophoresis. In ovarian cancer cell lines and others, RNA interference was also tested for and collected for Western Blot analysis—a process in which cells are electrophoresed to separate a mixture of proteins based on molecular weight; the only antibody that remains is that of the protein of interest [45,46]. Finally, after conducting the assays and protein-separation procedures, statistical analysis is used in each research paper to present the mean and standard deviations of the results. The mean results of the control samples and the drug samples are then tested using a one-way analysis of variance (ANOVA), followed by a two-tailed test (*p* <.05 results in a statistically significant difference from the control samples, indicating that the drug can potentially be used against this cancer) [47].

### 3.2. In vitro Cancer Cell lines

**Figure 2:**
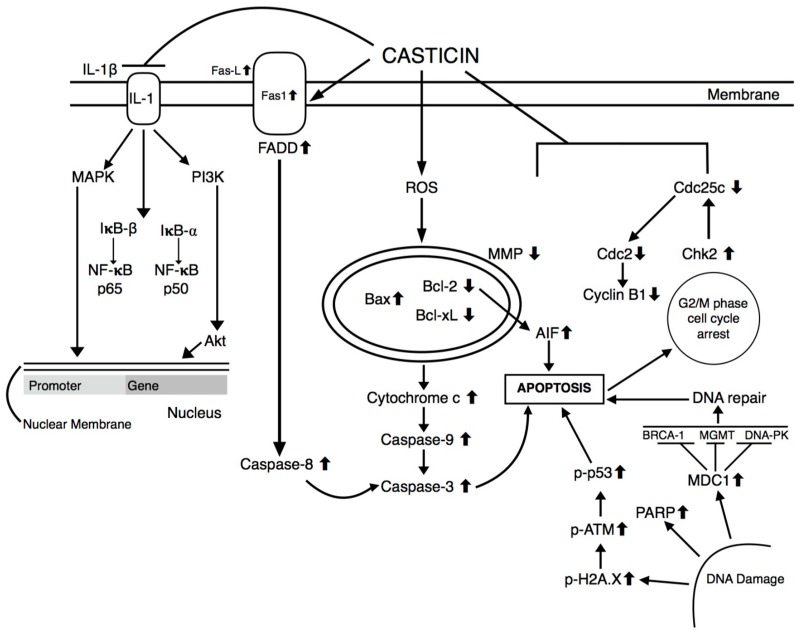
Molecular pathways affected by casticin.

**Figure 3:**
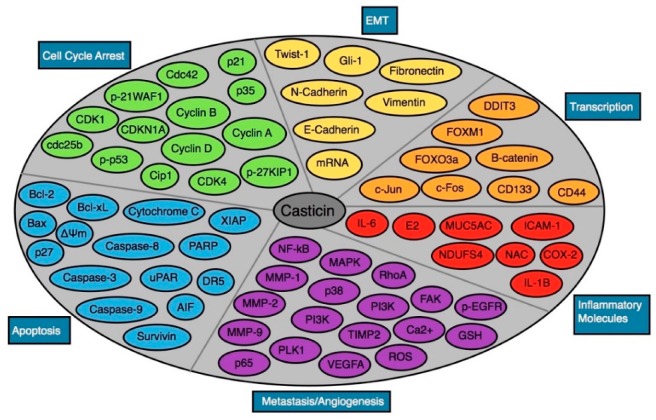
Different proteins modulated by casticin.

Casticin possesses antineoplastic activities in various cancer cell lines conducted in vitro, including breast, bladder, cervical, colon, esophageal, gallbladder, lung, oral, ovarian and prostate cancers, as well as hepatocellular carcinoma, leukemia and melanoma. Below is a brief summary of the specific effects of casticin on each of the aforementioned malignancies. Casticin is a novel phosphatidylinositol 3-kinase (PI3K) inhibitor, which is the predominant mechanism for its anticancer activity. Casticin can also upregulated in the Bax/Bcl-2 ratio through an increase in ROS generation in the mitochondria, as discussed in detail below [48]. The IC50 values, or half-maximal inhibitory concentrations, for available cell lines are also given. A visual representation of the specific molecular pathways targeted by the casticin is illustrated in Figure 2, while the various proteins affected by casticin are illustrated in Figure 3.

#### 3.2.1. Casticin and Breast Cancer

Breast cancer is the most prevalent type of cancer in women and is a major cause of mortality worldwide due to its poor prognosis and diagnostic procedures [49,50,51,52]. Pretreatment of casticin on MDA-MB-231 and 4T1 cells resulted in downregulation of MMP-9 mRNA protein expression but has no significant effect on MMP-2, suggesting that casticin can suppress both migration and invasion abilities in breast cancer cells. Although both proteins have similar properties, MMP-9 is directly involved in response to invasion, whereas MMP-2 is found only in malignant tumors, so it is not involved in initial invasive events. Within the AP-1 pathway, casticin suppressed c-Jun and c-Fos expression, leading to an increase in proliferative properties. Casticin also decreased the levels of phosphorylated Akt in the Akt signaling pathway. The reduction in MMP-9 protein levels reserved the invasion abilities of the breast cancer cell lines. The findings in this particular research paper suggest that casticin has properties to inhibit the PI3K/Akt signaling pathway, preventing cell invasion and migration in various tumor cells, including breast cancer [48]. The Akt signaling pathway is crucial for regulating both tumorigenesis (the formation of a tumor) and the cell cycle. The pathway is activated by a large number of receptor tyrosine kinases—for example, epidermal cell growth factor receptor (EGFR) and insulin-like growth factors (IFGRs)—with protein kinase B (PKB) being the central node that can be stimulated [48,53,54]. Casticin significantly inhibits cell viability by suppressing Akt activation in a concentration-dependent manner and also inhibits self-renewal and invasionary behaviors of cancer cells. Besides opioid receptors, casticin may also directly bind to several proteins, including estrogen receptors, threonine-protein kinase Pim-1 and reticuline oxidase, as revealed by docking studies [48,53]. As a result, casticin could be utilized as an antineoplastic drug for human breast cancers.

#### 3.2.2. Casticin and Bladder Cancer

Among men, bladder cancer is within the top eight leading causes of cancer death, with approximately 17,000–18,000 people being affected by bladder cancer each year [55]. An in vitro study conducted by Huang et al. demonstrated the ability of casticin to decrease the cell viability of bladder cancer TSGH-8301 cells through the induction of DNA damage and impairing DNA repair. Results indicated that the effect of casticin was more prominent after 48 h of treatment than after 12 h of treatment. After 48 h of treatment, casticin selectively inhibited p-ATM, p-ATR, MDC1 and MGMT levels. Contrastingly, casticin increased the levels of poly ADP-ribose polymerase (PARP) expression and upregulated p-p53 and p-H2A.X. Moreover, casticin also inhibited the translocation of DNA-PCKs and p-p53 proteins to the nucleus of the bladder cancer cell line [56].

#### 3.2.3. Casticin and Cervical Cancer

Following breast cancer, cervical cancer is the second deadliest deadly cancer among women [57,58]. Consequently, two studies have been conducted on the HeLa, CasKi and SiHa cervical cancer lines. Casticin induced cell cycle arrest in the sub-G1 phase and increased the reactive oxygen species (ROS) concentration within all three cell lines. Additionally, casticin induced mitochondrial apoptosis by the upregulation of Cytochrome c, the downregulation of MMP and an increase in caspase-3 and -9 activities. Bax was upregulated, while Bcl-xL and XIAP were both downregulated. Both mitochondrial signaling pathways and ROS production mediated the apoptosis. Mitochondrial-mediated apoptosis is a dissipation of the mitochondrial membrane potential and is modulated by the Bel-2 family of proteins. Contrastingly, ROS-mediated apoptosis is the result of the excess production of ROS, which induced oxidative damage and depolarized MMP. Doses of casticin between 0.5–4.0 µM were tested, with an IC50 value of 1.268 µM [59]. Another similar study also found that casticin increased levels of ROS production and increased the expression of phosphorylated JNK and c-Jun protein [60].

#### 3.2.4. Casticin and Colon Cancer

After lung and breast cancer, colorectal, or colon cancer, is the third-leading cause of cancer deaths in Western countries [61]. Casticin induced cell morphological changes and induced apoptosis via the activation of the caspase- and mitochondrial-dependent signaling cascade. Caspases are cysteine-aspartic protease enzymes which play a crucial role in inflammation, apoptosis, necrosis and other programmed cell death strategies. In caspase-mediated apoptosis, caspases catalyze the specific cleave factors of several regulatory proteins. Casticin upregulated ROS production but decreased Ca2+ ion levels, as well as the mitochondria membrane potential (ΔΨm). Casticin also downregulated intracellular protein targets such as cyclin-dependent kinase inhibitor 1A (CDKN1A), p21 and Cip1 proteins, whereas cAMP-responsive element-binding protein (CREB1), CDKN1B, p27 and Kip1 protein levels were increased. MMP-2 genes were inhibited by casticin in colon cancer cells. G2/M cell cycle arrest was also induced in Colo 205 cells when cells were incubated with 40 µM of casticin between 12–24 h. The results obtained are in agreement with other papers, which suggests that G2/M cell cycle arrest is induced by the increase of p27 expression. Additionally, casticin also increased levels of caspase-3, -8 and -9—however, only at a 12-h incubation period [62].

#### 3.2.5. Casticin and Esophageal Cancer

Although esophageal cancer does not have a high incidence rate relative to other cancers, it has an extremely high recurrence rate [12]. Most people diagnosed with esophageal cancer are simultaneously diagnosed with terminal cancer due to the aggressive metastatic ability of the neoplasm. Human esophageal cancer cell lines TE-11 and ECA-109 were tested in a dose-dependent fashion in vitro. Results indicated that casticin reduced levels of antiapoptotic Bcl-2 and increased levels of proapoptotic Bax proteins. Similar to colon cancer, casticin also upregulated levels of caspase-3 and -9, as well as PARP. This was mediated by the c-Jun N-terminal kinase (JNK) signaling pathway. It is important to note that the casticin samples used in this study were pretreated with a JNK pathway inhibitor, SP600125, which may have minimized the effect of the compound itself on the cell line. The levels of MMP were downregulated, while the levels of mitochondrial cytochrome c were increased. Cytochrome c acts as an electron carrier in the electron transport chain. In apoptosis, cytochrome c binds to cardiolipin, stimulating ROS-mediated apoptosis. In esophageal cancer cell lines, casticin inhibited the proliferative abilities and the clonogenicity (the ability of a cell to clone itself). Cell cycle arrest in sub-G1/G2 was also induced. Moreover, the same study discovered the antitumor properties of casticin in mouse xenograft models [63].

#### 3.2.6. Casticin and Gallbladder Cancer

Similar to esophageal cancer, gallbladder cancer (GBC) has the ability to spread to other lymph nodes in distant organs at a rapid rate, which, unless detected early, is fatal for patients diagnosed [64]. A study conducted on human NOZ and SGC996 gallbladder cell lines highlighted the ability of casticin (IC50 value: 2 µM) to induce cell cycle arrest in the G0/G1 phase and mitochondrial-dependent apoptosis in a dose-dependent manner in GBC cell lines. Bax and Bcl-2 are necessary components of mitochondrial-mediated pathways, which display a balance in normal cell growth, division and death. In cancer cells, Bcl-2 expression is increased, while Bax expression is contrastingly decreased. Casticin served to upregulate proapoptotic Bax protein and inhibit antiapoptotic Bcl-2 expression in NOZ and SGC996 cell lines. Additionally, casticin increased levels of caspase-3 and -9 and cleaved caspase-PARP. ΔΨm decreased in a dose-dependent manner, while p27 protein was upregulated, and cyclin-dependent kinase (CDK) 4 and phosphorylated protein kinase (PPK) B levels were decreased. An increase in p27 decreases p-Akt expression in the PI3K/Akt signaling pathway [65]. The study suggests casticin serves as an effective treatment for GBC.

#### 3.2.7. Casticin and Hepatocellular Carcinoma

Hepatocellular carcinoma (HCC) is a highly aggressive liver malignancy. Commonly, it occurs in people with pre-existing liver diseases, such as cirrhosis [66,67,68,69,70,71,72,73,74,75]. Many studies have been carried out on the effect of casticin on liver cancer stem cells (LCSCs) derived from the HCC cell cline. One such study conducted on the MHCC97 cell line found that casticin (IC50 value: 17.9 µM and 0.5 µM for parental cells and LCSCs, respectively) inhibited proliferative properties in a dose-dependent matter through the downregulation of B-catenin and blocking the Wnt/β-catenin pathway; the pathway regulates stem cell self-renewal and is present in liver cancer [76]. Another study evidenced the ability of casticin to induce apoptosis and G2/M cell cycle arrest in HepG2 and PLC/PRF/5 cells. Casticin silenced the forkhead box class O (FOXO) 3a phosphorylation protein by siRNA and decreased the expression of forkhead box M1 (FoxM1). FoxM1 is a proliferation-associated transcription factor and a downstream target of FOXO3a. The compound also inhibited other genes consisting of CDK1, cdc25B and cyclin B, while it upregulated p27/KIP1 expression. The FOXO3a/FoxM1 signaling pathway is, therefore, a viable pathway for the casticin molecule in HCC [77]. Moreover, casticin induced apoptosis of human HCC HepG2 and PLC/PRF/5 cells by increasing levels of caspase-3, -8 and -9 and downregulating glutathione (GSH) content in another study. Casticin (potency: 26.8% at 24 h) induced sub-G1 cell cycle arrest with a higher potency than 5-fluorouracil (potency: 17.4% at 24 h), a clinically tested cancer drug. It also increased death receptor (DR) 5 protein levels, which were inhibited by the thiol antioxidant acetylcysteine (NAC) [78]. In a fourth HCC study, casticin upregulated E-cadherin expression levels and decreased levels of N-cadherin in LCSCs in LCSC CD133+ cells. For 48 h of treatment, casticin attenuated epithelial-mesenchymal transition (EMT)-associated transcription factor Twist1 levels. The upregulation of Twist promotes N-cadherin and inhibits E-cadherin, so through downregulation of Twist1, casticin served to be an effective treatment against HCC LCSC cell lines [79].

#### 3.2.8. Casticin and Leukemia

Being ten times more prevalent in adults than children, leukemia is globally known to be the most common type of blood cancer. It affects essential components of the lymphatic system, including the bone marrow, blood cells and lymph nodes [6,80,81]. Two studies on the effect of casticin on leukemia treatment have been conducted in vitro. In HL-60 leukemia cells, casticin (IC50 value: 0.29 µM for 24 h and 1.15 µM for 48 h) induced apoptosis starting at 0.25 µM in a dose-dependent manner. Pretreatment with casticin reduced cell viability as the dose increased. However, for inducing G2/M cell cycle arrest, the optimal concentration was 0.3 µM. Casticin induced apoptosis by activating caspase-3, -8 and -9, inducing phospho-histone (H3) phosphorylation, upregulating intracellular ATP levels, increasing ROS and blocking the p38 mitogen-activated protein kinases (MAPK) pathway. This pathway is involved in inducing apoptosis by various stimuli in both normal human and cancer cells and can be activated by a variety of cellular factors, including ultraviolet light, inflammatory cytokines and proliferative factors. The study also found that the cytotoxicity of casticin acts independently of the ROS generation. Furthermore, pretreatment with casticin induced G2/M cell cycle arrest and displayed a significant reduction of cells in the G0/G1 and S phases [82]. In another study, casticin played a significant role in activating the PI3K/Akt signaling pathway through activating caspase-3 and PARP in HL-60, Kasumi-2 and K562 human leukemia cells. The resulting concentration of casticin was 5.95 µM, 4.82 µM and 15.56 µM for K562, HL-60 and Kasumi-1 cells, respectively. Moreover, there was significant accumulation of cells in the G2/M phase at 12 h, which gradually declined towards 48 h, suggesting the maximal activity of casticin in this study occurred at 12 h. The compound also upregulated P21waf1, P27kip1 and AV-positive PI-negative cells. Cell death in leukemia cell lines via apoptosis and mitotic catastrophe (an activator of the PI3K/Akt pathway) were induced, as well as G2/M cell cycle arrest [83]. These results collectively suggest that casticin poses as a strong therapeutic agent against leukemia treatment.

#### 3.2.9. Casticin and Lung Cancer

Lung cancer is the most commonly occurring cancer and causes more deaths worldwide than any other cancer. For metastasized lung cancers, the survival rate is only 5% [84,85,86,87]. Subsequently, there are numerous studies on the effectiveness of casticin on human lung cancer cell lines. Casticin downregulated uPAR and CD33, upregulated AMPK and ACC and silenced FOXO3 expression through the AMPK/FOXO3 signaling pathway in small-cell lung cancer (SCLC) H446 cells [88]. Another study isolated A549 cells from lung cancer stem-like cells (LCSLCs) from non-SCLC A549 cells, in which casticin reduced expression of CD133, CD44 and ALDH1 proteins and also suppressed MMP-9 activity. The compound inhibited proliferation in a dose-dependent manner, with an IC50 value of 14.3 µM and 0.4 µM on A549 and parental cells, respectively. Additionally, casticin has the ability to attenuate self-renewal and invasion in A549 cells, and there was downregulation of the Akt phosphorylation pathway [89]. Additionally, casticin downregulated IL-6 tumor necrosis factor α and suppressed cyclooxygenase 2 (COX-2) and prostaglandin E2 production. There was reduced mucin 5AC (MUC5AC), inflammatory cytokine levels and chemokine gene expression. The PI3K/Akt and MAPK both blocked the transcription of the NF-κB p65 protein in interleukin-1B (IL-1B) pulmonary epithelial cells [90]. Casticin blocked proinflammatory pathways and reduced existing inflammation in A549 lung cancer cells. Results showed an increase in MMP, cytochrome C protein and activated caspase-9 and -3. Proapoptotic Bax was upregulated, and the antiapoptotic X-linked inhibitor of apoptotic protein (XIAP) and B cell lymphoma-extra-large (Bcl-xL) were downregulated. Casticin-induced apoptosis was mediated through mitochondrial pathways and the upregulation of DR5; however, there was no significant effect on the death receptor (DR) 4 [91]. The compound targets various pathways in combating lung cancer; clinical tests are required to assess the effectiveness in patients.

#### 3.2.10. Casticin and Melanoma

Melanoma is a type of cancer that develops from melanocytes—a type of cell that contains melanin pigment. Melanomas typically prevail in the skin, but some rare forms of melanoma can occur in the mouth, eyes and even intestines [92]. An in vitro study conducted on mouse melanoma B16F10 cells examined gene expression levels of cell migration and invasion. It was detected that there were decreased MMP-1, MMP-2 and MMP-9 in the mitochondria, as well as downregulation of the NF-κB pathway, AKt pathway, FAK, p-EGFR, p-JNK and Rho A. Casticin increased the expression of certain genes, including cell adhesion molecule 1 (SCN1B), and decreased the expression of vascular endothelial growth factor A (VEGFA), which are associated with cell invasion and migration in mouse melanoma cell lines. Cell viability in melanoma cells decreased in a dose-dependent manner [93]. In another study, it was found that, in human melanoma A365.S2 cells, casticin induced morphological changes and mitochondrial-dependent apoptosis, damaged and condensed DNA and decreased the total number of viable cells. Casticin also induced G2/M cell cycle arrest by increasing the expression of p35, p31 and CHK-1 proteins, while downregulating Cdc25c, CDK1, cyclin A and cyclin B to control the cell cycle. Moreover, casticin inhibited the activation of tumorigenic NF-κB pathway [94] and increased ROS generation and caspase-3. A decrease in ΔΨm aided in mitochondrial-dependent apoptosis [95]. Consequently, casticin suits the purpose of being a potential chemo-preventive agent in melanoma skin cancer treatment in the future.

#### 3.2.11. Casticin and Oral Cancer

In 2018, around 350,000 cases of oral cancer were diagnosed worldwide, resulting in an estimated 170,000 deaths. It is a serious cancer and the most commonly occurring malignancy in the head and neck regions [96,97,98]. Casticin downregulated β-catenin, an essential component of the Wnt/β-catenin pathway, in human oral squamous cell carcinoma (OSCC) UM1 and HSC-3 cells. The overexpression of β-catenin induces proliferation, invasion and migratory activities in tumor cells, while its downmodulation can negatively regulate these tumorigenic processes. Additionally, casticin reversed epithelial-mesenchymal transition (EMT) in a dose-dependent manner [99]. In a second study, casticin inhibited cell viability by upregulating ROS and Ca2+ ions, decreasing ΔΨm; activating caspase-3, -8 and -9 and upregulating AIF and cytochrome c. Casticin also has the ability to inhibit EMT transitioning, where epithelial cells become more migratory by losing their polarity and junctions. Treatment with casticin induced cell morphological changes, condensed and damaged DNA and enabled G2/M cell cycle arrest in SCC-4 cells [100]. Casticin can therefore be tested as a potential antineoplastic agent towards OSCC cells in humans.

#### 3.2.12. Casticin and Ovarian Cancer

Being the most common type of cancer arising from gynecological tumors, one of the major treatments to ovarian cancer involves an oophorectomy or removing a woman’s ovaries entirely; this leads to forced menopause, depression, anxiety and premature death. Moreover, most patients with ovarian cancer who do not undergo surgery relapse within 18 months [101]. In a study conducted on SKOV3 and A2780 human ovarian cancer cells, it was concluded that casticin induced apoptosis and significantly reduced the expression of the FoxM1 gene. The effect of the compound resulted in reduced FOXO3a expression, along with downstream regulatory target genes, including surviving PLK1 and p27. Concentrations tested were 2.5 µM, 5.0 µM and 10.0 µM for 24 h, and it was found that casticin induced apoptosis through the inactivation of the FoxM1 gene [47]. Another similar study conducted on SKOV3 cells indicated that casticin upregulates E-cadherin and downregulates N-cadherin in ovarian cancer. Glioma-associated oncogene-1 (Gli-1) and EMT-associated hedgehog factors, including Twist1 levels, were also reduced. The hedgehog signaling pathway transforms key information necessary for cell differentiation [102]. In this way, casticin can inhibit cell invasion, migration and viability in human ovarian cancer cell lines.

#### 3.2.13. Casticin and Prostate Cancer

Prostate cancer is the third-most fatal cancer in developing countries and the second-most diagnosed cancer after lung cancer. Prostate cancer can also occur in a nonresponsive androgen-insensitive form, so it is vital that other drugs against prostate cancer are developed [103,104,105]. Casticin induced apoptosis and accompanied cell cycle arrest in the G2/M phase in human PC-3 cell lines. Casticin upregulated proapoptotic Bax, downregulated Bcl-2 and upregulated Cytochrome c, decreasing ΔΨm. The compound decreased cyclin B expression and CDK1; there was also an overproduction of ROS generations that depolarized MMP. In prostate cancer PC-3 cells, the IC50 value of casticin was reported to be 28.7 µM, suggesting that the compound is relatively less potent towards this type of cancer [106]. As a result, casticin serves as a viable chemo-preventive in prostate cancer. Table 1 summarizes the reported anticancer effects of casticin in in-vitro studies. 

### 3.3. In vivo Studies: Mouse Models

The effects of casticin have been tested in a great number of in vitro studies; nevertheless, even with a small number of studies conducted in vivo (performed on a live organism), the drug still showcased significant antineoplastic effects. The majority of in vivo experiments are performed on a xenograft model, which is a tissue/organ transplant that is derived from a different species and injected into the recipient—usually, a mouse. Casticin inhibited proliferation and induced apoptosis in a mouse xenograft model against esophageal cancer [63]. Casticin also prevented cadherin switching from taking place and aided cells in maintaining their normal migratory behavior in a mouse CD133+ cell line against hepatocellular carcinoma by upregulating E-cadherin and decreasing N-cadherin levels [79]. In a leukemia WEHI-3 mouse neoplasm, casticin increased macrophage phagocytosis from peripheral blood mononuclear cells (any peripheral blood cell containing a round nucleus—for example, T cells, B cells, NK cells and monocytes), causing cell endocytosis and eventual death [107]. Additionally, casticin induced mitochondrial-dependent apoptosis in response to myriad stimuli in a mouse melanoma model and also induced early G2/M cell cycle arrest [95]. Cancer cells undergo a “mitosis out-of-control” process to divide, adhering to their undifferentiated and proliferating nature. This means that cancer cells continue to divide without the regulation of cyclin-dependent kinases (CDKs). The increase of CDKs and the induction of cell cycle arrest at any stage can be analyzed using the cell cycle arrest apoptosis assay [41]. Table 2 summarizes the reported anticancer actions of casticin in preclinical models.

## 4. Effect of Casticin on Epithelial-Mesenchymal Transition (EMT)

EMT has been established as one of the key players in the tumorigenic process. It is characterized by increasing the expression of mesenchymal genes such as fibronectin, vimentin and N-cadherin and suppressing the expression of epithelial genes such as ocludin and E-cadherin [108,109,110,111,112,113,114,115]. It has been found that various concentrations of casticin can reverse the EMT process by increasing E-cadherin expression while inhibiting N-cadherin in oral squamous cell carcinoma (OSCC) UM1 cells [99], ovarian cancer SKOV3 cells [103] and liver cancer stem cells (LCSCs) derived from the SMMC-7721 cells [79]. After exposure to casticin, mRNA levels of Twist1 and N-cadherin decreased with the time progressed, most notably at 24 h in SKOV3 cells [103]. Twist overexpression in LCSCs attenuated casticin-induced regulation of E-cadherin and N-cadherin protein expression, as well as EMT capacity [79]. Like this, casticin exhibits therapeutic potential by inhibiting cell proliferation and EMT in many types of cancer cells.

## 5. Effect of Casticin on Metastasis

**Figure 4:**
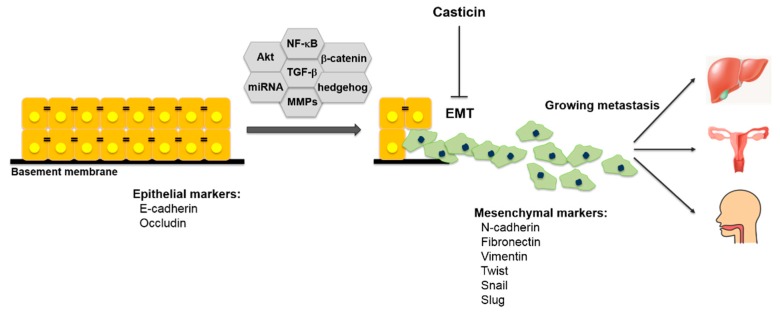
The role of casticin in regulating epithelial-mesenchymal transition (EMT).

Metastasis is the process by which cancer cells migrate throughout the body. The invasion of cancer cells into surrounding tissues and vessels is an early stage of tumor metastasis. As more than 90% of cancer patients die due to persistent recurrence, metastasis and chemotherapy resistance, there is a need for studies to prevent or target metastasis [116,117,118,119,120,121,122]. Casticin significantly reduced the cell migration rate by 77.41% after 48 h of 5 µM compared to the control of DU145 cells [99] and inhibited cell migration by 93% after 24 h of 200 nM in human melanoma A375.S2 cancer cells [123]. In addition, MMP-2/-9 activities were inhibited in a concentration- and time-dependent manner in DU145 cells [124]. Moreover, casticin significantly inhibited the motility ability of MDA-MB-231 and 4T1 cells [51]. The cDNA microarray analysis showed that casticin affected gene expression associated with cell migration and invasion, and DNA-damage-inducible transcript 3 (DDIT3) decreased four-fold in the group treated with casticin compared to the control group in B16F10 cells [93]. The role of the compound in regulating EMT is depicted in Figure 4.

## 6. Other Important Pharmacological Actions

For years, casticin has been used in Chinese folk medicine to alleviate premenstrual symptoms in women. A study conducted by Webster et al. investigated compounds extracted from V. agnus-castus, such as casticin, and their opioidergic mechanisms for premenstrual syndrome treatment. Casticin can bind to and activate µ- and δ-opioid receptor subtypes in a dose-dependent manner, reducing premenstrual syndrome effects. Casticin was reported to have therapeutic effects through the upregulation of these receptors [125]. Additionally, it is established that casticin displays anti-inflammatory properties. In an in vivo study conducted on female BALB/c mice, casticin reduced oxidative stress in the lungs of mice with asthma, alongside reduced activity by the aryl hydrocarbon receptor (AHR) and goblet cell hyperplasia. Moreover, casticin downregulated the levels of proinflammatory Th2 cytokine and eotaxin, resulting in reduced lung inflammation. It was reported that casticin is also a powerful immunomodulatory therapeutic drug and could be used in asthma treatment [126].

## 7. Cytotoxicity Data

The cytotoxicity data of casticin has been analyzed for in vitro and in vivo studies. Casticin reduced tumor growth rate and volume in all the in vivo studies discussed in this review article. In nude mice injected with esophageal cancer cells, casticin treatment was most effective at a dose of 10 mg/kg, causing a significant reduction in tumor weight. The control mice had a larger tumor volume and weight of 510.83 ± 94.95 mg, while both these factors significantly decreased with casticin dosages of 2 mg/kg and 10 mg/kg, where the mean weight of the mice was 369.83 ±70.04 mg and 193.50 ± 33.73 mg, respectively. There was no visible effect of apoptosis or necrosis on normal cells, whereas casticin induced apoptosis in the xenograft mouse model [63]. In leukemia WEHI-3 mice, pretreatment of casticin for a two-week period resulted in decreased weights of the liver and spleen at a dose of 0.4 mg/kg casticin. There was no significant difference in the appearances or body weights of the mice upon administration of casticin. T cell proliferation at 0.1, 0.2 and 0.4 mg/kg and B cell proliferation at 0.1 mg/kg were promoted, along with an increase in phagocytic activity from peripheral blood mononuclear cells (PBMCs). The highest dose of casticin, at 0.4 mg/kg, had a higher survival rate than ATRA, a preexisting leukemic drug. Additionally, all mice treated with 0.1 and 0.4 mg/kg of casticin survived [107]. In a concentration-dependent fashion, casticin had the ability to reduce the viability of A549 lung cancer cells at all concentrations of casticin tested, i.e., 1 µM, 5 µM and 10 µM [90]. Finally, casticin significantly reduced tumor volume and weight in an in vivo melanoma experiment. The mean weight of the tumors in control mice were three times greater than those treated with casticin at 2 mg/kg and 10 mg/kg. There was negligible difference in the overall body weights of the mice in both the casticin-treated and control groups [95]. In the case of all these models, casticin has the ability to suppress tumor growth without significantly affecting the weight of mice, suggesting the compound to be a viable anticancer treatment in preclinical studies.

## 8. Limitations and Future Prospects

Although various studies have been conducted on the potential anticancer effects of casticin on different cell lines, the majority of these studies are confined to in vitro studies. Only a handful of in vivo studies on cancers such as esophageal cancer, HCC, leukemia and melanoma have been conducted to assess the antineoplastic effects of casticin. Moreover, pharmacokinetic properties of casticin, including administration, bioavailability, distribution, etc., are limited due to the small number of in vivo studies conducted. One previous study has been conducted to quantify casticin dosages in rat plasma; the absolute bioavailability of casticin was reported to be 45.5% ± 11.0%, through both oral and intravenous methods of administration [127]. More such studies should be conducted to validate these figures in different types of cancers. Additionally, the safety and efficacy of casticin have not been reported as yet, which poses as a risk to assess the drug clinically. More studies on the pharmacokinetic properties of casticin can be investigated. In order to test the effectiveness and therapeutic applications of casticin in cancer patients, it is essential that clinical trials are conducted.

## 9. Conclusions

Cancer continues to persist as one of the deadliest diseases worldwide, responsible for the deaths of millions each year. Despite advancements in cancer research, the treatments may not always be effective, and they often occur alongside severe side effects. It is therefore essential to devise a safe and effective means to treat cancer. A suitable approach would be through the use of natural compounds, such as casticin. This review summarizes the antineoplastic effects of casticin, a flavonoid isolated from the Vitex species. Casticin has the ability to induce mitochondrial-dependent and ROS-mediated apoptosis, induce cell cycle arrest and inhibit the proliferation of invasive and migratory properties in breast, bladder, colon, lung, oral and ovarian cancers and others. Furthermore, casticin affects diverse oncogenic pathways, such as the MAPK, NF-κB and PI3K/Akt pathways, through the modulation of various proteins; increases ROS generation through the enhancement of Bax proteins and the decrease of Bcl proteins and inhibits cell division cycles (cdc25c and cdc2) and cyclins (B1) to induce cell cycle arrest. Moreover, in in-vivo studies, casticin was used to examine tumor growth and metastatic suppression in mouse models. Although casticin has displayed strong cytotoxic activities towards cancerous cells in preclinical studies, interestingly, pretreatment with casticin did not result in apoptosis or necroptosis of normal cells [62]. In fact, casticin aided in maintaining normal migratory behaviors of noncancerous cells in mice with hepatocellular carcinoma through the process of cadherin switching, thus serving as an effective anticancer agent [78]. Although many preclinical studies have been conducted in cell lines as well as mouse models to assess the anticancer effects of casticin, clinical trials are essential to report the therapeutic uses of casticin. More preclinical studies can be conducted to assess the pharmacokinetic properties of novel anticancer drugs, especially casticin. This may open new perspectives for the development of natural compounds as therapeutic drugs towards cancer treatments.

## Figures and Tables

**Table 1:** Antineoplastic effects of casticin under in vitro settings. ΔΨm: mitochondria membrane potential.

Cancer	Cell Line	IC50 Values	Phenotypic Effects	Mechanisms of Action	Ref
Breast Cancer	MDA-MB-231, 4T1 cells		Inhibited migratory activity and invasion	↓ MMP-9, ↓ c-Jun, ↓ c-Fos, ↓ Skt, ↑ MMP-2	[51]
Bladder Cancer	TSGH-8301		Induces DNA damage and impairs DNA repair	↓ p-ATM, ↓ p-ATR, ↓ MDC1, ↓ MGMT, ↑ p-p53, ↑ p-H2A.X, ↑ PARP	[56]
Cervical Cancer	HeLa, CasKi, SiHa, PBMC’s	1.268 µM	Accumulation of sub-G1 cells and induced mitochondrial apoptosis	↑ Cytochrome c, ↓ MMP, ↑ caspase-3, ↑ caspase-9, ↑ ROS, ↑ Bax, ↓ Bcl-xL, ↓ XIAP	[59]
	HeLa, CasKi, SiHa		Induced apoptosis	↑ ROS, ↑ JNK, ↑ c-Jun	[60]
Colon Cancer	Colo 205 cells		Induced apoptosis and altered associated gene expression	↑ ROS, ↑ caspase-3, ↑ caspase-8, ↑ caspase-9, ↓ ΔΨm, ↓ Ca^2+^, ↓ CDKN1A, ↓ p21, ↓ Cip1, Cdc42	[62]
Esophageal cancer	TE-1, ECA-109		Sub-G1/G2 cell cycle arrest, Induced EC apoptosis and inhibited proliferation and clonogenogenicity	↓ Bcl-2, ↑ Bax, ↑ caspase-3, ↑ caspase-9, ↑ PARP	[63]
Gallbladder Cancer	SGC996	2 µM	Induced mitochondrial-related apoptosis and G0/G1 cell cycle arrest	↑ Bax, ↓ Bcl-2, ↑ p27, ↓ cyclinD/CDK4, ↓ ΔΨm	[65]
Hepatocellular Carcinoma	LCSC’s HCC MHCC97		Inhibited proliferation	↓ B-catenin	[69]
	HepG2, PLC/PRF/5	17.9 µM for parental cells and 0.5 µM for LCSCs	Inhibited cell viability and colony formation and induced apoptosis G2/M cell cycle arrest	↓ FOXO3a, ↓ FoxM1, ↓ CDK1, ↓ cdc25B, ↓ cyclin B, ↑ p27/KIP1	[76]
	HepG2, PLC/PRF/5		Induced apoptosis and increased sub-G1 population	↑ Histone/DNA, ↑ caspase-3, ↑ caspase-8, ↑ caspase-9, ↓ GSH, ↓ DR5, ↑ NAC	[77]
	CD133+		Overexpression of Twist and cadherin switching	↑ E-cadherin, ↓ N-cadherin, ↓ EMT associated Twist-1	[78]
Leukemia	HL-60	0.29 µM for 24 h and 1.15 µM for 48 h	Induced apoptosis and G2/M cell cycle arrest	↑ caspase-3, ↑ caspase-8, ↑ caspase-9, ↑ H3 phosphorylation, ↑ intracellular ATP, ↑ ROS, ↓ MAPK	[79]
	HL-60, Kasumi-2, K562 cells		Induced apoptosis and mitotic catastrophe via PI3K/Akt pathway	↑ P21waf1, ↑ P27kip1, ↑ AV-positive PI-negative cells, ↑ PARP, ↑ caspase-3	[82]
Lung cancer	SCLC H446		Induced apoptosis	↓ uPAR, ↓ CD33, ↑ AMPK, ↑ ACC	[83]
	A549		Suppressed self-renewal and invasion of lung cancer stem-like cells (LCSLCs)	↓ CD133, ↓ CD44, ↓ ALDH1, ↓ MMP-9	[88]
	A549	14.3 µM for A549 cells and 0.4 µM for parental cells	Blocked proinflammatory cytokine and reduced inflammation	↓ IL-6, ↓ COX-2, ↓ E2, ↓ MU5AC, ↓ ICAM-1, ↓ proinflammatory cytokine, ↓ chemokine gene expression, ↓ MAPK, ↓ NF-κB p65	[89]
	H460, A548, H157		Induced caspase-mediated apoptosis	↑ MMP, ↑ Cytochrome c, ↑ caspase-9, ↑ caspase-3, ↑ Bax, ↓ XIAP, ↓ Bcl-XL	[90]
Melanoma	B16F10		Inhibits gene expression of cell migration and invasion	↓ MMP-9, ↓ MMP-2, ↓ MMP-1, ↓ FAK, ↓ 14–3-3, ↓ GRB2, ↓ Akt, ↓ NF-κB p65, ↓ SOS-1, ↓ p-EGFR, ↓ p-JNK 1.2, ↓ Rho A, ↑ SCN1B, ↑ TIMP2, ↓ NDUFS4, ↓ VEGFA, ↓ DDIT3	[91]
	A365.S2		Induced apoptosis and morphological changes, condensed and damaged DNA, decreased cell viability and induced G2/M cell cycle arrest	↑ ROS, ↑ caspase-3, ↓ ΔΨm, ↑ p35, ↑ p21, ↑ CHK-1, ↓ Cdc25c, CDK1, ↓ Cyclin A, ↓ Cyclin B, ↓ NF-κB p65	[94]
Oral cancer	UM1 and HSC-3		Inhibited cell viability, invasion and migration	↓ B-catenin	[95]
	SCC-4		Induced apoptosis and G2/M cell cycle arrest, decreased cell viability and condensed and damaged DNA	↑ ROS and Ca2+, ↓ ΔΨm, ↑ caspase-3, ↑ caspase-8, ↑ caspase-9, ↑ AIF, ↑ Cytochrome C	[100]
Ovarian cancer	SKOV3, A2780		Induced apoptosis through the loss of FoxM1	↓ PLK1, ↓ survivin, ↑ p27	[47]
	SKOV3		Reduced SKOV3 cell viability, migration and invasion	↓ Gli-1, ↓ Twist-1, ↓ N-cadherin, ↑ E-cadherin	[102]
Prostate Cancer	PC-3	28.7 µM	Induced apoptosis and G2/M cell cycle arrest	↑ Bax, ↓ Bcl-2, ↑ Cytochrome c, ↓ ΔΨm, ↑ ROS	[106]

**Table 2:** Antineoplastic effects of casticin under in vivo settings.

Cancer	Cell Line	Phenotypic Effects	Mechanisms of Action	Ref
Esophageal cancer	Mouse xenograft model	Inhibited proliferation and induced apoptosis	↓ Bcl-2, ↑ Bax, ↑ caspase-3, ↑ caspase-9, ↑ PARP	[63]
Hepatocellularcarcinoma	Mouse CD133+	Overexpression of Twist and cadherin switching	↑ E-cadherin, ↓ N-cadherin, ↓ EMT associated Twist-1	[79]
Leukemia	Mouse WEHI-3	Increased macrophage phagocytosis from peripheral blood mononuclear cells (PBMCs)	↑ NK cells cytotoxic activity, ↑ T-cells	[107]
Melanoma	Mouse A375.S2 xenograft model	Early-G2/M cell cycle arrest and induced mitochondrial apoptosis	↓ Tumor volume	[95]

## Abbreviations

AHR	Aryl hydrocarbon receptor
Bcl-2	B cell lymphoma-2
Bcl-xL	B cell lymphoma-extra large
Bax	BCL2-associated X protein
CD44	Cluster of differentiation 44
CDK1	Cyclin-dependent kinase 1
CDK4	Cyclin-dependent kinase 4
CDKN1A	Cyclin-dependent kinase inhibitor 1A
CDKN1B	Cyclin-dependent kinase inhibitor 1B
COX-2	Cyclooxygenase 2
CREB1	cAMP responsive element binding protein
DDIT3	DNA-damage-inducible transcript 3
DR4	Death receptor 4
DR5	Death receptor 5
EGFR	Epidermal cell growth factor receptor
EMT	Epithelial-mesenchymal transition
FOXO3a	Forkhead box class O 3a
FoxM1	Forkhead box M1
GBC	Gallbladder cancer
Gli-1	Glioma-associated oncogene 1
GSH	Glutathione
HCC	Hepatocellular carcinoma
IFGR	Insulin-like growth factors
IL-6	Interleukin 6
IL-B	Interleukin B
JNK	c-Jun N-terminal kinase
LCSC	Liver cancer stem cells
MAPK	Mitogen-activated protein kinases
MMP	Matrix metalloproteinase
MMP-1	Matrix metalloproteinase 1
MMP-2	Matrix metalloproteinase 2
MMP-9	Matrix metalloproteinase 9
MUC5AC	Mucin 5AC
NAC	Acetylcysteine
NDUFS4	NADH dehydrogenase (ubiquinone) Fe-S protein 4
NF-κB	Nuclear factor kappa light-chain enhancer of activated B cells
OSCC	Oral squamous cell carcinoma
PARP	Poly ADP-ribose polymerase
PBMC	Peripheral blood mononuclear cell
PI3K	Phosphatidylinositol 3-kinase
PKB	Protein kinase B
PLK1	Polo-like kinase 1
ROS	Reactive oxygen species
SCN1B	Cell adhesion molecule 1
TIMP2	TIMP metallopeptidase inhibitor 2
Twist1	Twist basic helix-loop-helix transcription factor 1
VEGFA	Vascular endothelial growth factor A
XIAP	X-linked inhibitor of apoptosis protein
ΔΨm	Mitochondria membrane potential
